# Supplementary material for: Risk factors for loneliness: The high relative importance of age versus other factors
Source: PLoS One. 2020 Feb 11;15(2):e0229087. doi: 10.1371/journal.pone.0229087 (PMC7012443; doi:10.1371/journal.pone.0229087)
Supplement: S1 Table — (DOCX) [file pone.0229087.s001.docx]

|  | 10-15 Years Old (*N*=441) | 16 Years and Older (*N*=4,444) |
| --- | --- | --- |
| Cronbach’s alpha | .81 | .83 |
| Item 1 *M* (*SD*) | 2.06 (.73) | 2.01 (.74) |
| Item 2 *M* (*SD*) | 1.91 (.73) | 2.00 (.72) |
| Item 3 *M* (*SD*) | 2.00 (.79) | 1.94 (.77) |
| Item 1 Difficulty | .69 | .67 |
| Item 2 Difficulty | .64 | .67 |
| Item 3 Difficulty | .67 | .65 |
| Item 1 Sensitivity | .63 | .64 |
| Item 2 Sensitivity | .68 | .69 |
| Item 3 Sensitivity | .64 | .72 |
