# Supplementary material for: Risk factors for loneliness: The high relative importance of age versus other factors
Source: PLoS One. 2020 Feb 11;15(2):e0229087. doi: 10.1371/journal.pone.0229087 (PMC7012443; doi:10.1371/journal.pone.0229087)
Supplement: S2 Table — (DOCX) [file pone.0229087.s002.docx]

| Group |  | Pearson *r* | |
| --- | --- | --- | --- |
| 10-15 Years Old |  | Item 2 | Item 3 |
|  | Item 1 | .57 | .57 |
|  | Item 2 | - | .63 |
| 16 Years and Older |  |  |  |
|  | Item 1 | .56 | .60 |
|  | Item 2 | - | .67 |
