# Supplementary figures and images for: Risk factors for loneliness: The high relative importance of age versus other factors
Source: PLoS One. 2020 Feb 11;15(2):e0229087. doi: 10.1371/journal.pone.0229087 (PMC7012443; doi:10.1371/journal.pone.0229087)

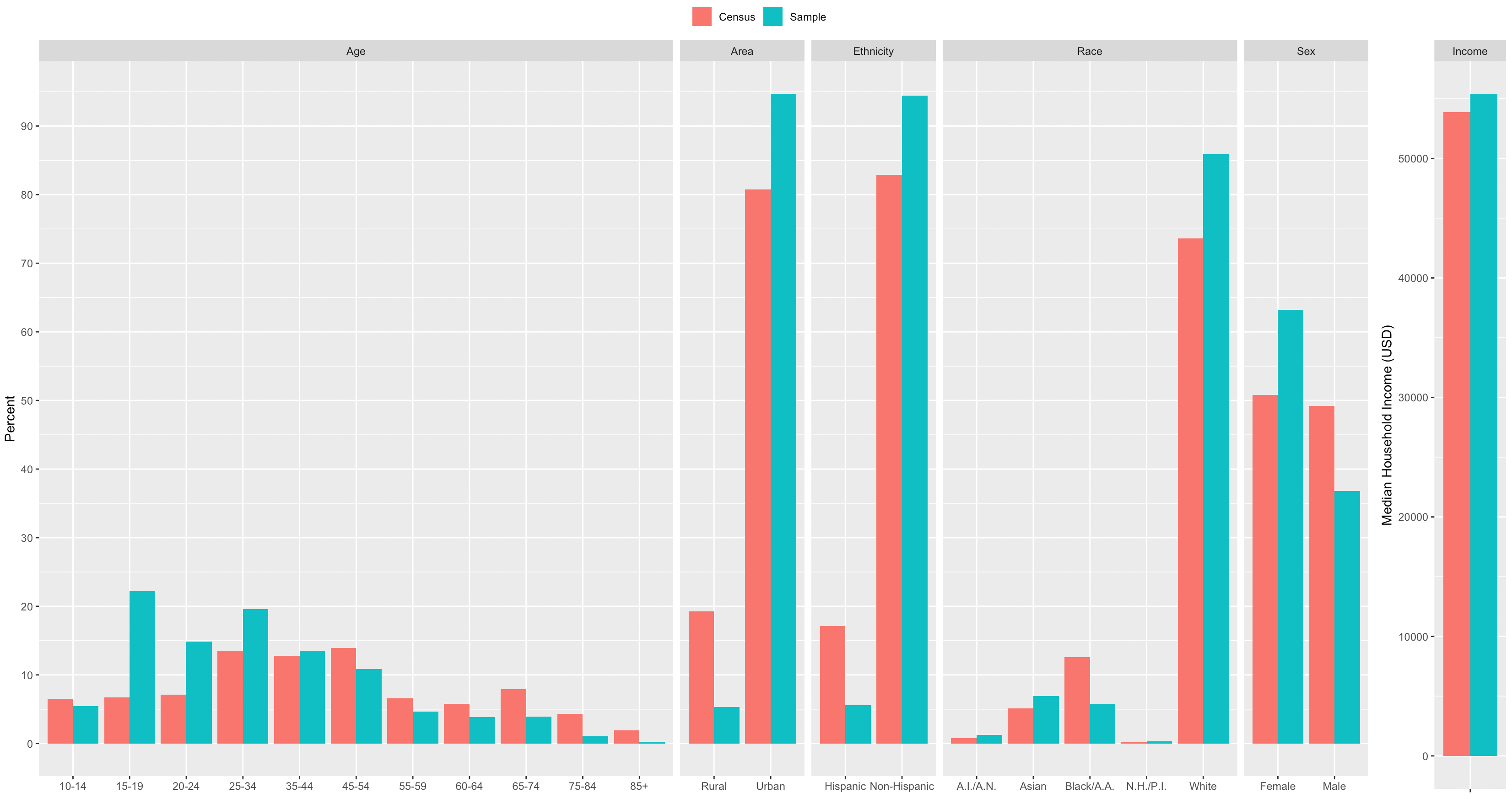

Supplement: S1 Fig — Data from the United States were retrieved from the Census 2011–2015 data. Data on urban versus rural areas are from the 2010 Census and based on definitions provided by the Census (i.e., Urban areas are those with populations of 2,500 or above). (TIF) [file pone.0229087.s003.tif]

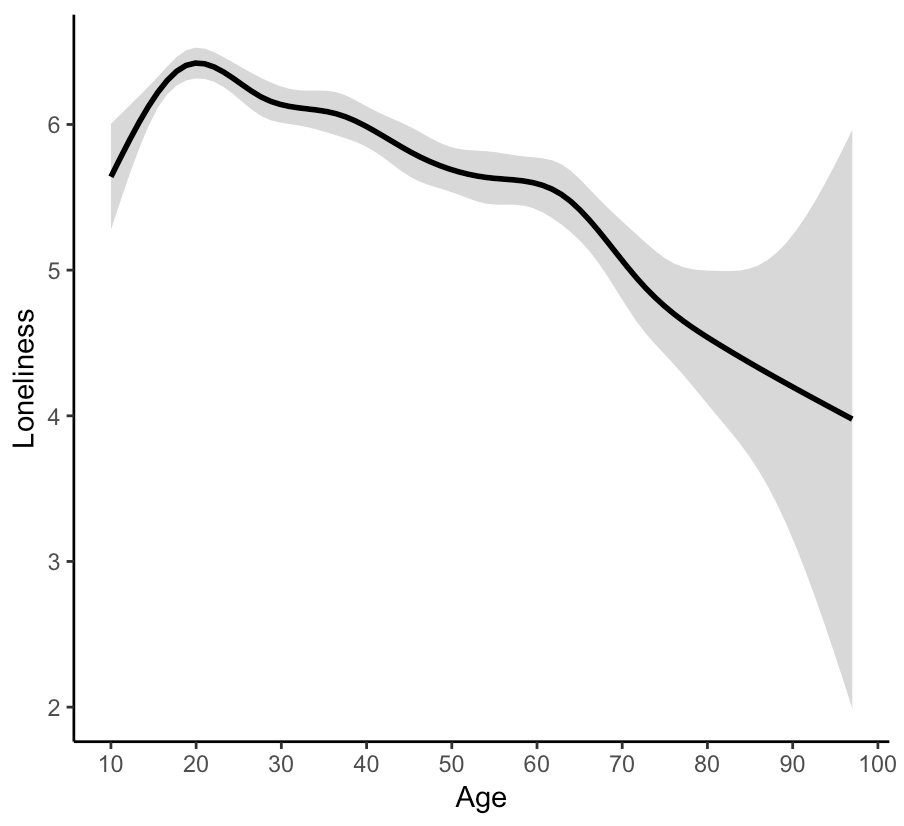

Supplement: S2 Fig — LOESS curve depicted with shaded area representing 95% CI. (TIF) [file pone.0229087.s004.tif]
